# Red Cell Distribution Width as a Prognostic Indicator in Pediatric Heart Disease and after Surgery

**DOI:** 10.1155/2014/681679

**Published:** 2014-03-12

**Authors:** Vural Polat, Sahin Iscan, Mustafa Etli, Helin El Kılıc, Özgür Gürsu, Esra Eker, Fatih Ozdemir

**Affiliations:** ^1^Department of Cardiovascular Surgery, Van Research and Education Hospital, 65100 Van, Turkey; ^2^Department of Anesthesiology and Reanimation, Van Research and Education Hospital, 65100 Van, Turkey

## Abstract

*Background*. Red cell distribution width (RDW) is an important marker which reflects inflammatory activity in many chronic diseases. The objective of this study is to investigate the impact of RDW on morbidity and mortality before and after pediatric congenital heart surgery. *Methods*. 107 patients with congenital heart disease, cardiac case group, and 70 patients, control group, without heart disease were retrospectively analyzed. Pre-, and postoperative and at discharge RDW of the cardiac patients were determined. Lengths of hospital and intensive care unit (ICU) stay and exited patients were determined. *Results*. Mean lengths of ICU and hospital stay were 3.3 ± 2.7 and 7.3 ± 2.9 days. In control group, mean preoperative RDW was 12.6 ± 1.4, while in cardiac case group it was significantly higher (15.1 ± 3.5). In cardiac case group, postoperative RDW were significantly higher than preoperative period, while RDW at discharge were significantly lower than postoperative estimates. A significant and a positive correlation was detected between lengths of ICU and hospital stay and RDW. RDW of the exited patients were significantly higher than the survivors. *Conclusions*. This study demonstrates that RDW can be used as an important indicator in the prediction of morbidity and mortality during pre-, and postoperative period of the pediatric congenital heart disease surgery.

## 1. Introduction

Congenital heart diseases (CHD) constitute an important cause of mortality and chronic diseases in the pediatric age. Medical and surgical treatment of CHD cause higher rates of morbidity and mortality in the pediatric age group [1, 2]. In these patients, inflammatory response and related markers increase especially in cases with advanced symptoms of heart failure [3]. This reaction occurring in patients with CHD develops along with inflammatory activation occurring preoperatively in myocardial tissues and postoperatively in parallel with inflammatory response taking place in all organs associated with cardiopulmonary bypass and postoperative stress. Severity of pre- and postoperative inflammatory reaction and oxidative stress is proportional to morbidity and mortality rates [1, 4].

The parametre of red cell distribution width (RDW) is indicative of variations in the sizes of red blood cells [5]. RDW has been used as an* auxiliary* marker in many diverse diseases, which progress generally with destruction and decrease in the production of erythrocytes. However, recent studies on RDW have demonstrated its relationship with inflammation and oxidative stress. In these studies, a direct correlation between cardiovascular diseases related to higher oxidative stress and increased RDW values has been detected. Besides, its value as a potential prognostic marker in the progression of heart failure has been demonstrated [6, 7].

The objectives of this study are to demonstrate preoperative significance of RDW as a simple and easily available indicator of inflammatory and oxidative stress in patients scheduled for surgery with the indication of CHD and its applicability as a predictive marker of both clinical status of the patient during postoperative follow-up period and in-patient morbidity and mortality.

## 2. Material and Methods

### 2.1. Study Design

This is a single-centered observational study. After obtaining approval of the Local Ethics Committee, among 107 pediatric cases which were operated in our clinic between January 2012 and August 2013 with the indication of CHD constituted the Cardiac Case group. Seventy age-matched patients without congenital heart diseases who consulted at our outpatient Clinics of Children's Health and Diseases within the same time interval comprised the control group. The patients in the control group did not have inflammatory or chronic disease either. These patients did not have chronic drug use. All patients in both groups were retrospectively analyzed.

Age, gender of all cases, and operations performed on the cardiac case group were recorded. Baseline RDW values were measured after overnight fasting using a Mindray BC-6800 automated hematology analyzer (Shenzhen, China). It was the same in the cardiac case and in the control group. Whole blood cell counts and biochemical values of the survivors in the cardiac case group were determined one day before (preoperative) and after (postoperative) the operation and before discharge from the hospital; while for dead cases these parameters were measured one day before their death. All of the patients which are in the cardiac case group were operated using cardiopulmonary bypass. Red blood cell transfusions in the operating room and later at the ICU were determined in the cardiac case group. In the control group, WBCs of the patients were determined on admission to hospital. Lengths of intensive care unit (ICU) and hospital stay of the operated patients were estimated. In the cardiac case group, discharged and exited patients were ascertained.

There was no difference at performing of the medical treatment which may cause change on the RDW value in preoperative and postoperative period in the cardiac case group. Using of the anesthetic agents, antihypertensive agents, and postoperative antibiotherapy was not different among the patients.

### 2.2. Statistical Analysis

Data were analyzed using the Statistical Package for Social Sciences 19.0 for Windows (SPSS Inc., Chicago, IL). A normal distribution of the quantitative data was checked using Kolmogorov-Smirnov test. Parametric tests were applied to data of normal distribution and nonparametric tests were applied to data of questionably normal distribution. Independent Samples* t-*test,* chi-*square test, and Mann-Whitney *U* test were used for the comparison of cardiac case and the control groups with respect to age, gender, and whole blood cell counts. In the cardiac case group, for the comparison of the laboratory values estimated pre- and postoperatively and at discharge, Paired-Samples* t-*test and Wilcoxon test were used. For individual comparisons of RDW values of the survived and exited cardiac patients detected in all three measurement time points, Mann-Whitney *U* Test was used. Potential correlations between lengths of ICU and hospital stay and RDW values were performed using Spearman Correlation Analysis. All differences associated with a chance probability of .05 or less were considered statistically significant.

## 3. Results

A total of 107 (53 males (49.5%) and 54 females (50.5%); mean age, 5.0 ± 4.1 yrs) and 70 (40 males (57.1%) and 30 females (42.9%); mean age, 5.1 ± 3.1 yrs) patients were enrolled into the cardiac case and the control groups, respectively. In a total of 107 patients, various surgical operations were performed including repairs of ventricular (*n* = 42), atrial (*n* = 21), complete atrioventricular (*n* = 6) septal defects, tetralogy of Fallot (*n* = 26), and valvular defects (*n* = 12). Mean duration of ICU and hospital stay was 3.3 ± 2.7 and 7.3 ± 2.9 days in the cardiac case group. Nine operated patients died during ICU monitoring.

In the comparison of the control and cardiac case groups with respect to preoperative whole blood cell counts using Independent Samples *t*- and Mann-Whitney *U* tests, RDW values were found to be significantly higher in the cardiac case group (*P* < 0.05). In the control group, mean RDW value was 12.6 ± 1.4, while in the cardiac case group it was higher (15.1 ± 3.5). Comparative data related to other hematological parameters are given in Table 1.

Individual comparisons among RDW values estimated in the cardiac case group pre- and postoperatively and at the time of discharge using Wilcoxon test and Paired Samples *t*-test revealed significantly increased postoperative RDW values relative to preoperative estimates (*P* < 0.05). Besides, RDW values detected at the time of discharge were significantly lower when compared with the postoperative RDW measurements (*P* < 0.05). However a significant difference was not found between RDW values calculated preoperatively and at the time of discharge (*P* > 0.05). After comparison red blood cell transfusions in the cardiac case group, there was no significant difference related to red blood cell transfusions in the operating room and later at the ICU (*P* > 0.05). Comparative data related to whole blood cell counts and biochemical parameters are given in Table 2.

A significantly positive correlation was detected in the cardiac case group between lengths of hospital and ICU stay and RDW values estimated pre- and postoperatively and at the time of discharge as assessed by Spearman Correlation Analysis (*P* < 0.05) (Table 3). RDW values of the survived and exited patients estimated pre- and postoperatively and at the time of discharge were compared using Mann-Whitney *U* test and significantly higher RDW values were detected in exited patients (*P* < 0.05) (Table 4).

## 4. Discussion

RDW is a parameter, which demonstrates variations in the dimensions of circulating erythrocytes (i.e. anisocytosis). Routinely, the value of this marker can be learnt during a simple whole blood count. Up to now RDW values have been assessed in hematological indications as malnutritional anemias and diseases leading to the destruction of erythrocytes. However, thanks to recent investigations, RDW measurements are requested for a wider spectrum of indications [8].

Numerous studies have indicated RDW as an applicable parameter in the prediction of risk and determination of prognosis in cardiovascular diseases including heart failure, diseases of peripheral arteries and lungs, myocardial infarction, and angina pectoris [9–11].

Higher RDW value is a risk factor used for the determination of unfavourable outcomes of chronic heart failure [5, 12, 13]. Felker et al. [14] have demonstrated that in the prediction of mortality and morbidity of chronic heart failure, RDW is an important marker. They also indicated that RDW also complies with widely accepted risk assessment tools as ejection fraction, functional classification of heart failure, and renal functions with comparable statistical level of significance and concluded that RDW is a potential prognostic factor in heart failure.

Even though the mechanism of this important implication ascribed to RDW has not been fully determined, in some relevant studies theories related to oxidative stress and inflammatory reactions have led the way [6, 15, 16]. Because of their higher antioxidant capacity, erythrocytes are more vulnerable to the effects of oxidative damage. Oxidative stress and chronic inflammation decrease production of erythropoietin and aggravates destructive process of erythrocytes leading to ineffective production of red blood cells. Erythrocyte damage, decrease in the life cycle of available erythrocytes, and entrance of the immature erythrocytes into the general circulation increase levels of RDW [6, 8, 15]. Since inflammation and oxidative stress also play a role in the progression of cardiovascular diseases, RDW is thought to be an important marker in cardiovascular diseases [17]. The first outcome derived from this study is the finding of significantly higher RDW values in the cardiac patients scheduled for a surgical intervention because of CHD in comparison with the control group which consisted of pediatric patients without any heart disease. This finding suggests for us that higher RDW levels might stem from oxidative stress, inflammatory changes, and erythrocyte damage occurring because of an organic defect detected in patients with CHD scheduled for surgical intervention.

A number of studies also demonstrated higher RDW levels to be a potential predictive and prognostic marker in coronary and peripheral arterial diseases [11, 18, 19]. Tonelli et al. [8] investigated the relationship between RDW values and the risk of death due to all causes and development of cardiovascular event in coronary artery patients without heart failure and observed a significant correlation between the baseline RDW levels and all-cause mortality in patients followed up for 60 months. Besides, risk of newly-developed cardiovascular event and mortality was higher in patients with increased RDW values. In addition, an association between higher RDW levels on the one hand and newly-developed myocardial infarction and symptomatic heart failure and stroke was revealed. In a study, which investigated prognostic value of RDW following percutaneous coronary interventions, after an average of 4 years of follow-up, RDW was revealed as a robust predictive marker of mortality [20]. The second outcome derived from this study is related to the impact of RDW in the prediction of morbidity and mortality following pediatric heart surgery. In pediatric cases operated for CHD, postoperative RDW values were found to be significantly higher relative to preoperative estimates. While RDW values detected at the time of discharge were relatively lower than postoperative values. This finding supports the significance of RDW as a potentially important prognostic marker in congenital heart surgery in that RDW reveals the clinical status of a patient after a major operation such as congenital heart surgery, which exposes the patient to a greater amount of oxidative stress.

Warwick et al. [21] investigated the significance of RDW in patients candidate for coronary bypass surgery and analyzed in-hospital mortality, long-term survival rates, lengths of hospital, and ICU stay. They found that RDW is an important factor in the prediction of in-hospital mortality and long-term survival but an insignificant factor for the determination of lengths of ICU and hospital stay. In this study, a positive correlation was demonstrated between RDW values and lengths of ICU and hospital stay. This finding indicates that RDW as a potentially significant marker in the prediction of lengths of ICU and hospital stay. In addition, significantly higher RDW values detected in exited patients relative to survivors substantiate the applicability of RDW in the prediction of morbidity and mortality.

This study has some potential limitations. First of all, it is a single-centered retrospective study which included small number of patient population. Residual confounding factors might have thus affected the results, regardless of the adjusted analysis. Secondly, information about other clinical markers was not provided. Further studies explaining the mechanism of relationship between mortality and morbidity are needed.

In conclusion, RDW can be a preoperative marker in pediatric patients with CHD and an important parameter, which can be used in the prediction of patient's clinical state during the postoperative follow-up period. Even though more comprehensive studies which can explain the mechanism of the relationship between the results obtained and RDW values are needed, higher RDW levels can be used as an important indicator in the determination of lengths of postoperative ICU and hospital stay, in addition to in-hospital morbidity and mortality following pediatric congenital heart surgery.

## Figures and Tables

**Table 1:** Clinical characteristics of the patients and comparative data related to whole blood cell counts estimated for the cardiac case and the control groups.

	Patient group				Control group				*P*
	Mean ± SD/*n*—%		Median (Min−Max)		Mean ± SD/*n*—%		Median (Min–Max)		
Age	5.0 ± 4.1		4	0–18	5.1 ± 3.1		5	1–12	0.881 *t *
Gender									
Female		54—50.5%				* *30—42.9%			0.322 *χ* ^2^
Male		53—49.5%				* *40—57.1%			
Duration of ICU stay (days)	3.3 ± 2.7		3	1–25					
Duration of hospital stay (days)	7.3 ± 2.9		7	4–26					
WBC (10^9^/L)	9.7 ± 3.6		9	5–24	8.4 ± 2.4		8	4–17	**0**.**005** *t *
RBC(10^12^/L)	5.1 ± 3.7		5	3–42	4.8 ± 0.4		5	4–6	0.219 m
HGB (g/dL)	15.4 ± 24.9		13	7–270	14.0 ± 11.9		12	10–112	0.208 *t *
PLT (10^9^/L)	265.4 ± 91.5		252	5–529	313.1 ± 79.3		296	168–607	**0**.**000** m
RDW (%)	15.1 ± 3.5		14	9–36	12.6 ± 1.4		13	8–16	**0.000** m
MCV (fL)	81.2 ± 6.3		81	53–91	74.7 ± 7.6		75	25–86	**0.000** m

SD: Standard deviation; Min: minimum; Max: maximum; *t*: Independent *t*-test; *χ* ^2^: chi-square test; m: Mann-Whitney *U* test; ICU: intensive care unit; WBC: white blood cell; RBC: red blood cell; HGB: hemoglobin; PLT: Platelet; RDW: red cell distribution width; MCV: mean corpuscular volume.

**Table 2:** Comparative data related to pre- and postoperative hematological and biochemical values in the cardiac case group.

					Mean ± SD		
	Preoperative	Postoperative	Discharge		Preoperative/postoperative change	Preoperative/discharge change	Postoperative/discharge change
RDW (10^9^/L)	15.1 ± 3.5	16.0 ± 3.5	14.9 ± 2.7		0.9 ± 2.5	−0.3 ± 3.1	−1.1 ± 2.6
				*P*	**0**.**000**	0.918	**0**.**000** *w *
WBC (10^9^ /L)	9.7 ± 3.6	12.6 ± 4.1	11.3 ± 3.8		2.9 ± 4.3	1.6 ± 3.6	−1.3 ± 4.6
				*P*	**0**.**000**	**0**.**000**	**0**.**006** *e *
HGB (g/dL)	15.4 ± 24.9	13.5 ± 13.3	13.4 ± 6.6		−2.0 ± 11.8	−2.0 ± 18.6	−0.1 ± 7.0
				*P*	**0**.**000**	0.093	**0**.**001** *w *
RBC (10^12^/L)	5.1 ± 3.7	4.6 ± 3.1	4.7 ± 2.8		−0.5 ± 0.8	−0.4 ± 1.2	0.1 ± 0.7
				*P*	**0**.**000**	**0**.**000**	0.059 *w *
PLT (10^9^/L)	265.4 ± 91.5	165.2 ± 65.4	241 ± 130		−100.2 ± 94	−23.7 ± 134	76.5 ± 122.0
				*P*	**0**.**000**	**0**.**005**	**0**.**000** *e *
MCV (fL)	81.2 ± 6.3	83.4 ± 5.2	84.7 ± 4.1		2.2 ± 4.6	3.5 ± 5.8	1.3 ± 3.7
				*P*	**0**.**000**	**0**.**000**	**0**.**000** *e *
BUN (mg/dL)	23.8 ± 7.7	30.5 ± 11.3	26.7 ± 13		6.7 ± 11.9	2.9 ± 14.6	−3.8 ± 11.8
				*P*	**0**.**000**	0.157	**0**.**001** *w *
CRE (mg/dL)	0.33 ± 0.12	0.38 ± 0.17	0.40 ± 0.2		0.05 ± 0.13	0.07 ± 0.23	0.02 ± 0.16
				*P*	**0**.**000**	**0**.**011**	0.968 *w *
ALT (U/L)	18.6 ± 15.4	34.9 ± 59.0	82.7 ± 393		16.4 ± 51.7	64.2 ± 393.8	47.8 ± 374.8
				*P*	**0**.**000**	**0**.**000**	**0**.**048** *w *
AST (U/L)	37.4 ± 24.2	95.8 ± 105.0	127 ± 679		58.4 ± 101.0	89.9 ± 680.9	31.5 ± 639.2
				*P*	**0**.**000**	0.059	**0**.**000** *w *
ALB (g/dL)	4.32 ± 0.40	3.86 ± 0.43	4.0 ± 0.38		−0.46 ± 0.49	−0.27 ± 0.45	0.19 ± 0.42
				*P*	**0**.**000**	**0**.**000**	**0**.**000** *w *

SD: standard deviation; *w*: wilcoxon test; *e*: paired sample *t*-test (*P* < 0.005).

RDW: red cell distribution width; WBC: white blood cell; HGB: hemoglobin; RBC: red blood cell; PLT: Platelet; MCV: mean corpuscular volume; BUN: blood urea nitrogen; CRE: creatinine; ALT: alanine aminotransferase; AST: aspartate aminotransferase; ALB: albumin.

**Table 3:** Assessment of the correlation between RDW values measured pre- and postoperatively and at the time of discharge and lengths of ICU and hospital stay.

		RDW		
		Preoperative	Postoperative	Discharge
Duration of ICU stay (days)	*R*	0.257	0.342	0.361
	*P*	**0**.**008**	**0**.**000**	**0**.**000**
Duration of hospital stay (days)	*R*	0.291	0.385	0.394
	*P*	**0**.**002**	**0**.**000**	**0**.**000**

Spearman correlation (*P* < 0.005).

ICU: intensive care unit.

**Table 4:** Comparison of RDW values measured pre- and postoperatively and at the time of discharge in survived and exited patients.

	Alive			Exitus			*P* value
	Mean ± SD	Median (Min–Max)		Mean ± SD	Median (Min–Max)		
RDW							
Preoperative	15.0 ± 3.3	14	12–36	17.0 ± 4.6	16	9–26	**0.024**
Postoperative	15.7 ± 3.0	15	13–33	19.1 ± 5.9	19	8–30	**0**.**002**
Discharge	14.6 ± 2.3	14	11–26	17.8 ± 4.5	17	11–25	**0**.**009**

Mann-Whitney *U* Test (*P* < 0.005).

SD: standard deviation; Min: minimum; Max: maximum.
